# PPAR*α* Agonist WY-14643 Induces SIRT1 Activity in Rat Fatty Liver Ischemia-Reperfusion Injury

**DOI:** 10.1155/2015/894679

**Published:** 2015-10-11

**Authors:** Eirini Pantazi, Emma Folch-Puy, Mohamed Bejaoui, Arnau Panisello, Ana Teresa Varela, Anabela Pinto Rolo, Carlos Marques Palmeira, Joan Roselló-Catafau

**Affiliations:** ^1^Department of Experimental Pathology, Institute of Biomedical Research of Barcelona (IIBB-CSIC), IDIBAPS, Barcelona, 08036 Catalonia, Spain; ^2^Department of Life Sciences and Center for Neuroscience and Cell Biology, University of Coimbra, 3004-517 Coimbra, Portugal; ^3^Department of Biology, University of Aveiro, 3810-193 Aveiro, Portugal; ^4^Centro de Investigación Biomédica en Red de Enfermedades Hepáticas y Digestivas (CIBEREHD), Barcelona, 08036 Catalonia, Spain

## Abstract

Ischemia-reperfusion injury (IRI) remains a frequent complication in surgery, especially in case of steatotic livers that present decreased tolerance towards IRI. Apart from its major role in metabolism, activation of peroxisome proliferator-activated receptor *α* (PPAR*α*) has been related with positive effects on IRI. In addition, the deacetylase enzyme sirtuin 1 (SIRT1) has recently emerged as a promising target for preventing IRI, through its interaction with stress-related mechanisms, such as endoplasmic reticulum stress (ERS). Taking this into account, this study aims to explore whether PPAR*α* agonist WY-14643 could protect steatotic livers against IRI through sirtuins and ERS signaling pathway. Obese Zucker rats were pretreated or not pretreated with WY-14643 (10 mg/kg intravenously) and then submitted to partial (70%) hepatic ischemia (1 hour) followed by 24 hours of reperfusion. Liver injury (ALT levels), lipid peroxidation (MDA), SIRT1 activity, and the protein expression of SIRT1 and SIRT3 and ERS parameters (IRE1*α*, peIF2, caspase 12, and CHOP) were evaluated. Treatment with WY-14643 reduced liver injury in fatty livers, enhanced SIRT1 activity, and prevented ERS. Together, our results indicated that PPAR*α* agonist WY-14643 may exert its protective effect in fatty livers, at least in part, via SIRT1 induction and ERS prevention.

## 1. Introduction

Ischemia-reperfusion injury (IRI) is a limiting factor for the outcome of many clinical conditions and although the extensive investigations, the underlying mechanisms remain largely unclear [1]. Moreover, the increased rates of obesity have resulted in the augmented number of livers with severe steatosis [2]. Steatotic livers present an exaggerated accumulation of lipids which contributes to the activation of various cellular stress signaling pathways and finally to increased vulnerability against IRI [3]. Consequently, there is an augmented interest for identifying mechanisms able to reduce IRI in steatotic livers.

Peroxisome proliferator-activated receptor *α* (PPAR*α*) is a nuclear receptor that is highly expressed in metabolically active tissues, such as liver, and functions as a lipid sensor. Upon the binding of various lipids, it forms heterodimers with the retinoid X receptor (RXR) and activates the transcription of various genes that regulate lipid homeostasis and metabolism, including genes involved in mitochondrial *β*-oxidation, fatty acid uptake and binding, and lipoprotein transport [4]. In fact, PPAR*α* activation is associated with reduced hepatic steatosis [5, 6] through the regulation of a wide variety of genes involved in peroxisomal, mitochondrial, and microsomal fatty acid *β*-oxidation systems in the liver [7].

Various studies have evidenced the antioxidant and anti-inflammatory effects of PPAR*α* agonists against IRI in various organs; WY-14643 efficiently decreased neutrophil infiltration and proinflammatory cytokine expression (TNF-*α* and IL-1*β*) and prevented the formation of ROS [8–10]. In addition, PPAR*α* has also been associated with the prevention of endoplasmic reticulum stress (ERS), a common feature of IRI [11]. Pretreatment with PPAR*α* agonist WY-14643 protected liver HepG2 cells against ERS-induced apoptosis by downregulating the expression of BiP and C/EBP homologous protein (CHOP), two components of the ERS-mediated apoptosis pathway. Moreover, ERS has been linked to a number of downstream pathways that contribute to the pathogenesis of nonalcoholic fatty liver disease [12].

Sirtuin 1 (SIRT1), NAD^+^-dependent protein deacetylase, is involved in numerous physiological processes including cellular stress response, glucose homeostasis, and immune response. In accordance with its role as a metabolic mediator, SIRT1 is known to regulate genes involved in fatty acid oxidation and lipolysis [13]. Among them, PPAR*α* is a well-known factor that is activated by SIRT1 [14, 15]. SIRT1 deletion in hepatocytes impaired the activity of PPAR*α*, resulting in development of hepatic steatosis, whereas SIRT1 hepatic overexpression suppressed the expression of gluconeogenic genes and attenuated obesity-induced ERS [16, 17]. Furthermore, sirtuin 3 (SIRT3), another member of the sirtuin's family, has also been involved in metabolic regulation [18, 19] and both SIRT1 and SIRT3 have emerged as potential targets to diminish IRI [20].

This study aims to explore new mechanisms by which a PPAR*α* agonist, WY-14643, exerts its beneficial effects against hepatic IRI in a genetic model of obese rats. SIRT1 and ERS signaling appear to be potential targets of WY-14643.

## 2. Materials and Methods

### 2.1. Experimental Animals

Homozygous obese (Ob) Zucker rats (Charles River, France) aged 16 weeks were used; Ob rats lack the cerebral leptin receptor and are characterized by severe macro- and microvesicular fatty infiltration in hepatocytes. Animals had free access to water and standard laboratory food* ad libitum* and were kept under constant environmental conditions with a 12-hour light-dark cycle. All procedures were performed under isoflurane inhalation anesthesia. This study was performed in accordance with European Union regulations (Directive 86/609 EEC). Animal experiments were approved by the Ethics Committees for Animal Experimentation (CEEA, Directive 396/12), University of Barcelona.

### 2.2. Experimental Design

Rats were randomly divided into three experimental groups: (1) Sham, *n* = 6; (2) ischemia-reperfusion (IR), *n* = 6; and (3) WY-14643 + IR, *n* = 6. A model of partial (~70%) hepatic warm ischemia was applied. Briefly, a midline laparotomy was performed and the portal triad was dissected free of surrounding tissue. Then, an atraumatic clip was placed across the portal vein and hepatic artery to interrupt the blood supply to the left lateral and median lobes of the liver. After 60 min of partial hepatic ischemia, the clip was removed to recover hepatic reperfusion for 24 hours. Sham control rats underwent the same protocol without vascular occlusion. In the group of WY-14643 + IR, rats were treated with WY-14643 (10 mg/kg intravenously) 1 hour before the induction of IR [21]. After 24 h of reperfusion, rats were sacrificed; blood samples were drawn from aorta and ischemic lobes were collected and stored at −80°C until assayed.

### 2.3. Biochemical Determinations

#### 2.3.1. Transaminases Assay

Hepatic injury was assessed in terms of transaminases levels with a commercial kit from RAL (Barcelona, Spain). Briefly, blood samples were centrifuged at 4°C for 10 min at 3000 rpm and then were kept at −20°C. In order to assay transaminase activity, 200 *μ*L of the supernatant was added to the substrate provided by the commercial kit. ALT levels were determined at 365 nm with an UV spectrometer and calculated following the supplier instructions.

#### 2.3.2. Lipid Peroxidation Assay

Lipid peroxidation in liver was used as an indirect measurement of the oxidative injury induced by ROS. Lipid peroxidation was determined by measuring the formation of malondialdehyde (MDA) with the thiobarbiturate reaction. MDA in combination with thiobarbituric acid (TBA) forms a pink chromogen compound whose absorbance at 540 nm was measured. The result was expressed as nmols/mg protein.

#### 2.3.3. SIRT1 Activity Assay

SIRT1 activity was determined according to the method described by Becatti et al. with some modifications [22]. Protein extracts were obtained using a mild lysis buffer (50 mM Tris-HCl pH 8, 125 mM NaCl, 1 mM DTT, 5 mM MgCl_2_, 1 mM EDTA, 10% glycerol, and 0.1% NP40). SIRT1 activity was measured using a deacetylase fluorometric assay kit (CY-1151, CycLex, MBL International Corp.), following the manufacturer's protocol. A total of 25 *μ*L of assay buffer containing the same quantity of protein extracts (10 *μ*g/*μ*L) was added to all wells, and the fluorescence intensity was monitored every 2 min for 1 h using the fluorescence plate reader Spectramax Gemini, applying an excitation wavelength of 355 nm and an emission wavelength of 460 nm. The results are expressed as the rate of reaction for the first 30 min, when there was a linear correlation between the fluorescence and this period of time.

#### 2.3.4. ATP Quantification

Tissue samples (20 mg) were pulverized in liquid N_2_ and homogenized in ice-cold 25 *μ*L of KOH buffer (KOH 2.5 M, K_2_HPO_4_ 1.5 M). Homogenates were then vortexed and centrifuged at 14,000 ×g at 4°C for 2 min. The supernatants were collected and dissolved in 100 *μ*L of K_2_HPO_4_ 1 M. Following this, pH was adjusted to 7 and samples were frozen at −80°C for posterior use. Finally, adenosine nucleotides were quantified with an ATP bioluminescent assay kit (Sigma-Aldrich) on a Victor 3 (PerkinElmer, Waltham, MA) plate reader.

#### 2.3.5. NAD^+^/NADH Determination

Hepatic NAD^+^/NADH levels were quantified with a commercially available kit (MAK037, Sigma Chemical, St. Louis, MO, United States) according to the manufacturer's instructions.

### 2.4. Western Blotting Analysis

Liver tissue was homogenized in RIPA buffer (Tris-HCl pH = 7.5 50 mM, NaCl 150 mM, SDS 0.1%, C_24_H_39_O_4_Na 1%, NP-40 1%, EDTA 5 mM, Na_3_VO_4_ 1 mM, NaF 50 mM, and DTT 1 mM, 1 complete tablet/100 mL). Fifty *μ*g of proteins was electrophoresed on 8–15% SDS-PAGE gels and transblotted on PVDF membranes (Bio-Rad). Membranes were then blocked with 5% (w/v) nonfat milk in TBS containing 0.1% (v/v) Tween 20 and incubated overnight at 4°C with anti-SIRT1 (number 07-131, Merck Millipore, Billerica, MA); anti-SIRT3 (number 2627), anti-p-eIF2a (Ser51, number 9721), anti-IRE1*α* (number 3294), and anticaspase 12 (number 2202) were purchased from Cell Signaling (Danvers, MA), NAMPT (AP22021SU, Acris Antibodies GmbH, Germany), anti-GADD 153 (sc-575, Santa Cruz Biotechnology), and anti-GADPH (G9545, Sigma Chemical, St. Louis, MO, USA). After washing, bound antibody was detected after incubation for 1 h at room temperature with the corresponding secondary antibody linked to horseradish peroxidase. Bound complexes were detected using Western Bright ECL-HRP substrate (Advansta) and were quantified using the Quantity One software for image.

### 2.5. Statistical Analysis

Data are expressed as mean ± standard error. Statistical comparison was performed by variance analysis, followed by the Student-Newman-Keuls test, using the GraphPad Prism software. *P* < 0.05 was considered statistically significant.

## 3. Results

### 3.1. WY-14643 Administration Decreased Hepatic Injury and MDA Levels in Obese Rats

First of all, we aimed to investigate the effect of WY-14643 pretreatment on hepatic injury in obese rats. As shown in Table 1, IR group was associated with increased ALT levels, which was prevented after treatment with WY-14643 (Table 1). In addition, pretreatment with the PPAR*α* agonist decreased the release of lipid peroxidation products as observed for the low MDA levels (Table 2).

### 3.2. WY-14643 Treatment Increased SIRT1 Activity, While No Effects Were Found on SIRT1 and SIRT3 Protein Expression

**Figure 1:**
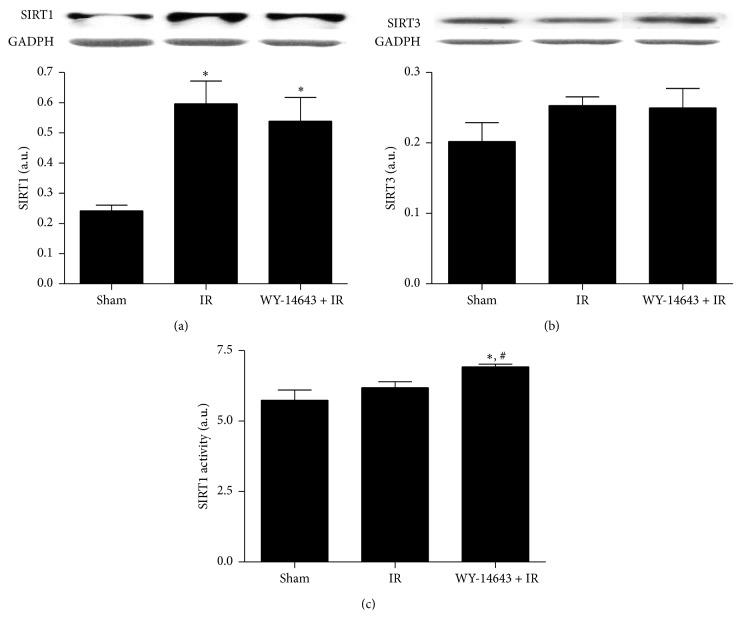
WY-14643 pretreatment and sirtuins protein expression and activity in steatotic livers after ischemia-reperfusion. Western blot and densitometric analysis of (a) SIRT1 and (b) SIRT3. (c) SIRT1 enzymatic activity. Sham: anesthesia and laparotomy, IR: 60 min partial ischemia and 24 h of reperfusion, and WY-14643 + IR: iv administration of WY-14643 (10 mg/kg) 1 hour before IR. ^*∗*^ *P* < 0.05 versus Sham; ^#^ *P* < 0.05 versus IR.

It is known that hepatic deletion of SIRT1 alters PPAR*α* signaling, but we then explored whether PPAR*α* activation could affect the protein expression of SIRT1 and SIRT3. No changes on SIRT3 protein expression were observed among all the experimental groups (Figure 1(b)). By contrast, although SIRT1 protein expression increased during ischemia-reperfusion, its levels were not significantly different between IR and WY-14643 pretreated rats (Figure 1(a)). In addition, WY-14643 treatment resulted in enhanced SIRT1 activity in comparison to both Sham and IR group (Figure 1(c)).

### 3.3. WY-14643 Administration Enhanced NAD^+^ Levels

**Figure 2:**
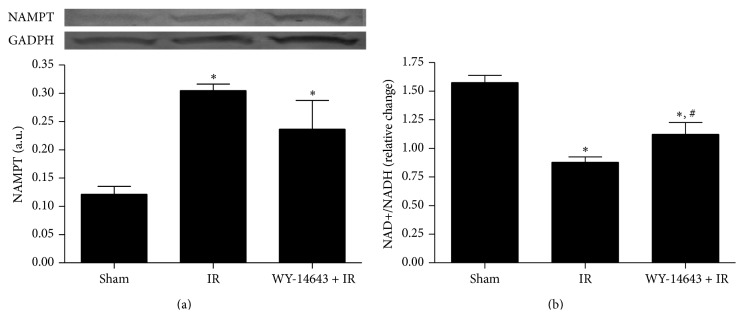
Effect of WY-14643 administration in NAMPT protein expression and NAD^+^/NADH levels. (a) Western blot and densitometric analysis of NAMPT. (b) Photometric analysis of NAD^+^/NADH levels in steatotic livers after 24 hours of reperfusion. Sham: anesthesia and laparotomy, IR: 60 min partial ischemia and 24 h of reperfusion, and WY-14643 + IR: iv administration of WY-14643 (10 mg/kg) 1 hour before IR. ^*∗*^ *P* < 0.05 versus Sham; ^#^ *P* < 0.05 versus IR.

Due to the fact that SIRT1 depends on NAD^+^ levels, we determined the NAD^+^/NADH levels and the protein expression of nicotinamide phosphoribosyltransferase (NAMPT), a well-known mediator of NAD^+^ biosynthetic pathways. As evidenced in Figure 2(a), both IR and WY-14643 + IR groups showed augmented NAMPT levels when compared to Sham group. Moreover, obese rats submitted to IR presented significant decreases of NAD^+^/NADH levels in contrast to untreated animals, but WY-14643 contributed to more elevated NAD^+^ levels than IR group (Figure 2(b)).

### 3.4. WY-14643 Pretreatment Augmented ATP Levels

**Figure 3:**
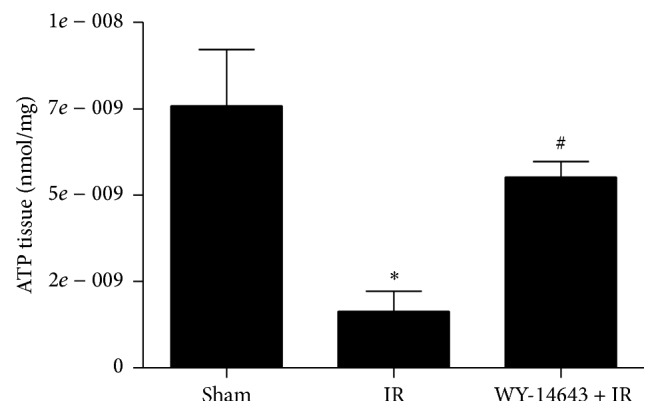
Role of WY-14643 treatment in ATP levels in steatotic livers subjected to ischemia-reperfusion. Sham: anesthesia and laparotomy, IR: 60 min partial ischemia and 24 h of reperfusion, and WY-14643 + IR: iv administration of WY-14643 (10 mg/kg) 1 hour before IR. ^*∗*^ *P* < 0.05 versus Sham; ^#^ *P* < 0.05 versus IR.

As PPAR*α* induces fatty acid oxidation which is a source of ATP production, we then measured ATP levels. We observed that IR significantly decreased ATP levels when compared to Sham group, whereas WY-14643 administration previous to IR provoked an overwhelming increase in ATP levels (Figure 3).

### 3.5. PPAR*α* Enhancement Decreased ERS

**Figure 4:**
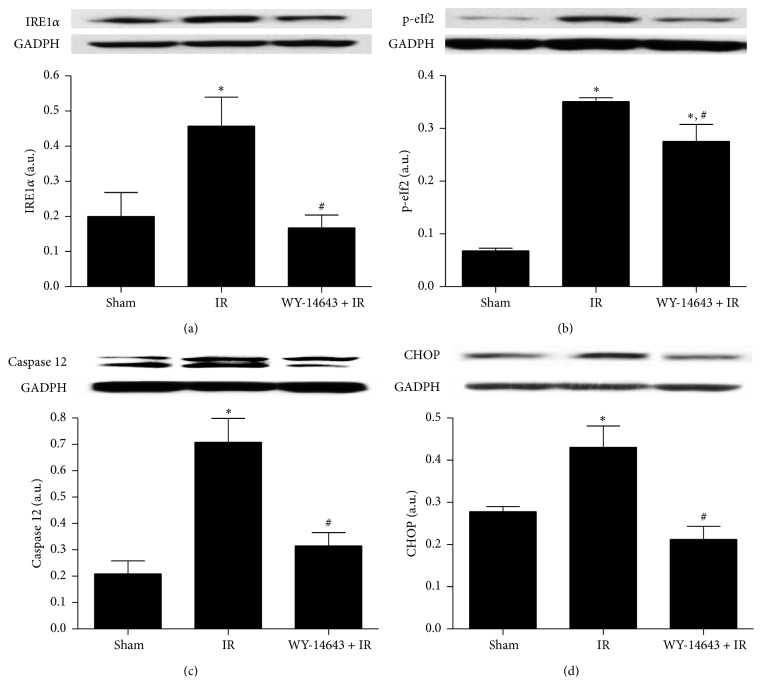
Implication of WY-14643 administration in ERS parameters in steatotic livers subjected to ischemia-reperfusion. Western blot and densitometric analysis of (a) IRE1*α*, (b) p-eIf2, (c) caspase 12, and (d) CHOP. Sham: anesthesia and laparotomy, IR: 60 min partial ischemia and 24 h of reperfusion, and WY-14643 + IR: iv administration of WY-14643 (10 mg/kg) 1 hour before IR. ^*∗*^ *P* < 0.05 versus Sham; ^#^ *P* < 0.05 versus IR.

Excessive lipid accumulation in the tissues has been associated with ERS induction [23]. Thus, possible alterations in protein expression of ERS parameters were evaluated. As shown in Figure 4, expression of IRE1*α*, p-eIF2, caspase 12, and CHOP was exacerbated by IR and restored by pretreatment with the PPAR*α* agonist WY-14643.

## 4. Discussion

PPAR*α* has gained special interest for its implication in metabolism and its protective effects in IRI models. However, the underlying interactions beyond its activation in obese livers subjected to IRI are not fully understood. In this study, we aimed to investigate the hepatoprotective mechanisms of PPAR*α* agonist WY-14643 in a genetic rat model of obesity.

To begin with, pretreatment of obese rats with WY-14643 proved to be protective against hepatic IRI. This result is consistent with other studies using genetic and dietary models of steatohepatitis. In nonalcoholic steatohepatitis (NASH) and simple steatosis, treatment of mice with WY-14643 protected steatotic livers against IRI [24]. Given the fact that oxidative injury is known to be more exaggerated in fatty livers [3, 25], we also determined lipid peroxidation and we found a significant reduction in MDA formation in the liver of rats pretreated with WY-14643. In this sense, PPAR*α* beneficial effects against oxidative stress have also been demonstrated in other models [26, 27].

Various studies have evidenced the protective role of SIRT1 against IRI, which in most cases has been associated with attenuation of oxidative stress [28, 29]. In the liver, a major target of SIRT1 is the PPAR*α* signaling pathway. Loss of hepatic SIRT1 impairs PPAR*α* mediated fatty acid metabolism and decreases fatty acid *β*-oxidation [17]. Additionally, cell culture experiments suggested that PPAR*α* may also positively regulate SIRT1 expression [30, 31]. In our study, we observed that WY-14643 administration did not affect either SIRT1 or SIRT3 protein expression, whereas it strengthened SIRT1 activity.

As SIRT1 requires NAD^+^ for its enzymatic activity, the augmented NAD^+^ levels that we observed in WY-14643 treated group led us to suggest that SIRT1 enhanced activity may be, at least in part, a result of the increased levels of its cofactor NAD^+^. However, the fact that NAD^+^ levels were not in accordance with NAMPT proteins levels may be attributed to the existence of other NAD^+^ precursors, like tryptophan and nicotinic acid [32].

It has been shown that PPAR*α* stimulates the *β*-oxidative degradation of fatty acids, provoking thus a high yield of ATP production [33, 34]. Indeed, in our study, we observed that administration of PPAR*α* agonist WY-14643 resulted in overwhelming ATP increases. Fatty livers are characterized by a reduced ATP content, which limits the resistance and the survival of hepatocytes against stress conditions, including IRI [3]. Thus, PPAR*α* activation diminished IRI in fatty livers by enhancing energy production. Furthermore, ATP forms part of the NAD^+^ biosynthesis pathways [35, 36] and in this way may promote SIRT1 activity.

Various studies have associated steatotic livers with ERS [12, 37]. IR affects the ability of the endoplasmic reticulum to synthesize and fold proteins, leading to the exaggerated accumulation of unfolded proteins and the initiation of ERS. Upon ERS, various proteins localized in the endoplasmic reticulum are activated, such as inositol requiring enzyme 1*α* (IRE1*α*) and pancreatic ER kinase- (PKR-) like ER kinase (PERK). Activated PERK phosphorylates eukaryotic initiation factor 2 (eIF2) in order to block protein synthesis and activated IRE1*α* controls genes involved in protein degradation. This consists of a cell attempt to restrain the accumulation of newly synthesized proteins in the ER lumen but can also initiate proapoptotic events, including the activation of caspase 12 and enhanced protein expression of CHOP [38]. Here, we observed that the IRE1*α*, p-eIF2, and CHOP signaling pathways of the ERS, as well as caspase 12 levels, were significantly abrogated after WY-14643 treatment. Due to the fact that ROS impairs the protein folding, the attenuation of oxidative stress by PPAR*α* activation could contribute to a more proper folding of the proteins and thus to lessened ERS. ERS prevention by PPAR*α* activation has also been evidenced* in vitro* in H_2_O_2_-treated HepG2 cells [11]. Another potential mechanism to prevent ERS might be based on the SIRT1 augmented activity, as SIRT1 hepatocyte loss has been shown to be crucial for the development of ERS in a high-fat diet [16].

## 5. Conclusion

In conclusion, our study gives a new insight into the hepatoprotective mechanisms of the PPAR*α* agonist WY-14643 in steatotic livers, implying that SIRT1 might be an important mediator of these beneficial effects. However, more efforts are required to elucidate the exact mechanisms that define the observed interactions.

## Figures and Tables

**Table 1:** Effect of WY-14643 administration in hepatic injury in steatotic livers subjected to ischemia-reperfusion.

ALT		
Sham	IR	WY-14643 + IR
97 ± 27,85	2675 ± 277,03^*∗*^	380 ± 86,02^*∗*,#^

Alanine aminotransferase (ALT) levels in plasma after 24 h of reperfusion. Sham: anesthesia and laparotomy, IR: 60 min partial ischemia and 24 h of reperfusion, and WY-14643 + IR: iv administration of WY-14643 (10 mg/kg) 1 hour before IR. ^*∗*^ *P* < 0.05 versus Sham; ^#^ *P* < 0.05 versus IR.

**Table 2:** Effect of WY-14643 on lipid peroxidation in steatotic livers subjected to ischemia-reperfusion.

MDA		
Sham	IR	WY-14643 + IR
0,32 ± 0,07	1,22 ± 0,13^*∗*^	0,76 ± 0,17^*∗*,#^

Photometric analysis of malondialdehyde levels (MDA). Sham: anesthesia and laparotomy, IR: 60 min partial ischemia and 24 h of reperfusion, and WY-14643 + IR: iv administration of WY-14643 (10 mg/kg) 1 hour before IR. ^*∗*^ *P* < 0.05 versus Sham; ^#^ *P* < 0.05 versus IR.
